# Alcohol and Apoptosis: Friends or Foes?

**DOI:** 10.3390/biom5043193

**Published:** 2015-11-19

**Authors:** Ana Rodriguez, Karan Chawla, Nsini A. Umoh, Valerie M. Cousins, Assama Ketegou, Madhumati G. Reddy, Mustafa AlRubaiee, Georges E. Haddad, Mark W. Burke

**Affiliations:** Department of Physiology & Biophysics, College of Medicine, Howard University, 520 W St., NW, Washington, DC 20059, USA; E-Mails: rodriguez.ani1108@gmail.com (A.R.); karan_078@hotmail.com (K.C.); nsini2k2@aol.com (N.U.); valeriecousins@gmail.com (V.C.); ak4000@nyu.edu (A.K.); mgr6k@virginia.edu (M.R.); mustafa.bakker@yahoo.co.uk (M.A.); ghaddad@howard.edu (G.H.)

**Keywords:** cardiomyocyte, apoptosis, alcohol, contractility, cardiomyopathy

## Abstract

Alcohol abuse causes 79,000 deaths stemming from severe organ damage in the United States every year. Clinical manifestations of long-term alcohol abuse on the cardiac muscle include defective contractility with the development of dilated cardiomyopathy and low-output heart failure; which has poor prognosis with less than 25% survival for more than three years. In contrast, low alcohol consumption has been associated with reduced risk of cardiovascular disease, however the mechanism of this phenomenon remains elusive. The aim of this study was to determine the significance of apoptosis as a mediating factor in cardiac function following chronic high alcohol *versus* low alcohol exposure. Adult rats were provided 5 mM (low alcohol), 100 mM (high alcohol) or pair-fed non-alcohol controls for 4–5 months. The hearts were dissected, sectioned and stained with cresyl violet or immunohistochemically for caspase-3, a putative marker for apoptosis. Cardiomyocytes were isolated to determine the effects of alcohol exposure on cell contraction and relaxation. High alcohol animals displayed a marked thinning of the left ventricular wall combined with elevated caspase-3 activity and decreased contractility. In contrast, low alcohol was associated with increased contractility and decreased apoptosis suggesting an overall protective mechanism induced by low levels of alcohol exposure.

## 1. Introduction

Alcohol abuse is one of the most relevant and disabling issues that plagues our society. It is the third leading preventable cause of death in the United States with an average of 79,000 casualties every year [1]. Results from the National Epidemiological Survey on Alcohol and Related Conditions performed in 2001–2002 show that 30.3% of adults in the United States have experienced alcohol use disorders at some point during their lives [2]. As a whole, excessive alcohol consumption cost an estimated $223.5 billion in 2006, a significant proportion of our total healthcare expenditures [1]. A major contributor of health-care expenses related to excess alcohol consumption is asymptomatic alcoholic cardiomyopathy (ACM) [3] which can lead to progressive heart failure with or without reduced ventricular wall thickness and cardiac output, accompanied with electrocardiogram (ECG) abnormalities [3,4].

Research in the field of cardiac health at the molecular level suggests that apoptosis is a major contributor in the occurrence of cardiovascular diseases [5,6,7]. Apoptosis is a regulated process of cell deletion that is characterized by events that result in nuclear and cellular fragmentation, as well as cell shrinkage [6]. It has been indicated that the proteolytic cleavage of key proteins by certain activated proteases known as caspases play a major role in the apoptotic pathways [8]. Evidence indicates that chronic alcohol exposure renders cells more prone to undergoing apoptosis [9]. This is commonly assumed to be a result of increased reactive oxygen species (ROS), which are important mediators in signal transduction during apoptosis of cardiomyocytes [10,11]. Due to the involvement of caspases in apoptosis it has been shown that overexpression of these enzymes leads to lethal cardiomyopathy in transgenic mice [9].

Despite these findings, recent studies have shown that moderate alcohol consumption may play a role in activating the PI3K/AKT pathway, thereby having cardioprotective effects [12]. The anti-apoptotic mechanism of alcohol remains elusive, but Yuan *et al.* [13], note that one possible explanation for this effect is the ability of the PI3K/AKT pathway to inhibit caspase-9 and negate its apoptotic function. Furthermore, our group [14,15] has recently shown that PI3K/AKT plays a crucial role in mediating the beneficial as well as detrimental cardiac effects of acute low and high doses of alcohol, respectively.

Myocardial damage is an important determinant of morbidity and mortality, and limiting the extent of cardiomyocyte apoptosis during oxidative stress has significant implications in therapeutics and cardiac health [6]. Our study aims to investigate the effects of high and low alcohol exposure on caspase-3 activity and its effect on contractility in rat hearts.

## 2. Results

**Figure 1:**
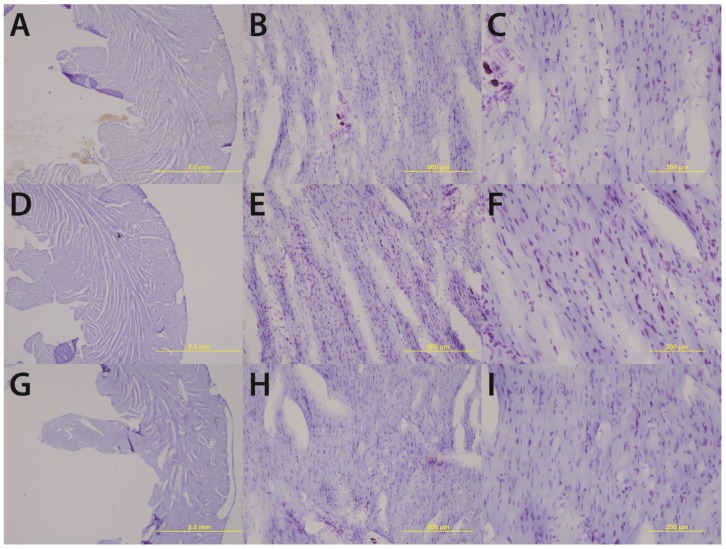
Chronic high alcohol (**G**–**I**) results in a thinning of the left ventricular wall accompanied by an enlargement of the ventricular lumen compared to age-matched and pair-fed chronic low alcohol (**D**–**F**) and control subjects (**A**–**C**). Magnifications of 1.25× (**A**, **D** & **G**) 10× (**B**, **E** & **H**) and 20× (**C**, **F** & **I**) are displayed above; scale bars = 5 mm, 500 μm, and 200 μm respectively.

**Figure 2:**
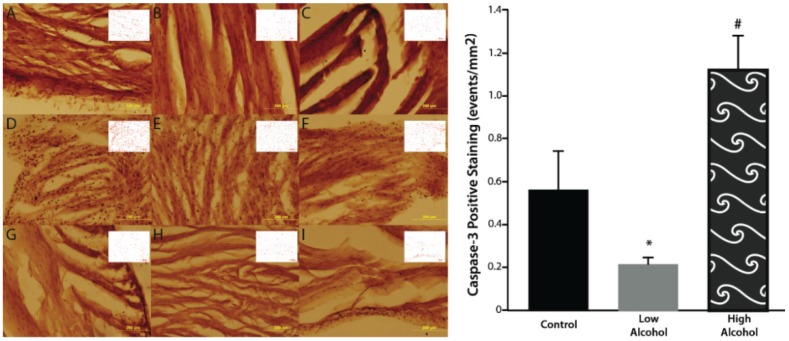
There is significantly elevated caspase positive events in the high alcohol group (**D**–**F**) compared to controls (**A**–**C**) and low alcohol animals (**G**–**I**) in the epicardial (**A**, **D**, **G**), myocardial (**B**, **E**, **H**), and endocardial (**C**, **F**, **I**) layers. Low alcohol (**G**–**I**) displayed significantly lower caspase positive events compared to both high alcohol (**D**–**F**) and controls (**A**–**C**). ImageJ-generated profiles are inset on each image. Magnification of 20× are displayed above; scale bar = 200 μm. * *p* < 0.05 compared to control and HA; # *p* < 0.05 compared to control and LA.

Gross histological observations suggest a thinning of the left ventricular wall of high-alcohol subjects compared to both control and low-alcohol subjects (Figure 1). Immunohistochemical analysis of caspase-3 levels indicated no significance between the epicardium, endocardium, and myocardium layers in the various treatment groups (high-alcohol F_(2,9)_ = 0.9032; *p* = 0.439; low-alcohol F_(2,9)_ = 3.825; *p* = 0.0628; control F_(2,9)_ = 1.032; *p* = 0.3948). Therefore to determine overall effects of treatment on caspase-3 levels, the data were collapsed across layers. Overall, there are significant differences between alcohol groups (F_(3,36)_ = 8.391; *p* = 0.0002) with high alcohol group displaying significantly more caspase-3 positive staining than control (*p* = 0.014) and low alcohol (*p* < 0.0001) groups (Table 1). The low alcohol group also had significantly less caspase-3 staining than the control group (*p* = 0.038; Figure 2).

**Table 1:** Chronic low alcohol subjects displayed significantly less caspase-3 events accompanied by an increased velocity shortening and peak cellular shortening compared to control subjects. Chronic high alcohol subjects displayed significantly elevated caspase-3 events compared to both control and chronic low alcohol subjects. * *p* < 0.05 compared to control; ^#^ *p* < 0.05 compared to LA and control.

	Control	LA	HA
Caspase-3 (events/mm^2^)	0.560 ± 0.187	0.216 ± 0.029 *	1.121 ± 0.154 ^#^
Velocity Shortening (µm/s)	89.6 ± 5.6	164.6 ± 17.1 *	99.65 ± 9.3
Peak Cellular Shortening (%)	5.3 ± 0.3	10.2 ± 1.2 *	6.2 ± 0.6

**Figure 3:**
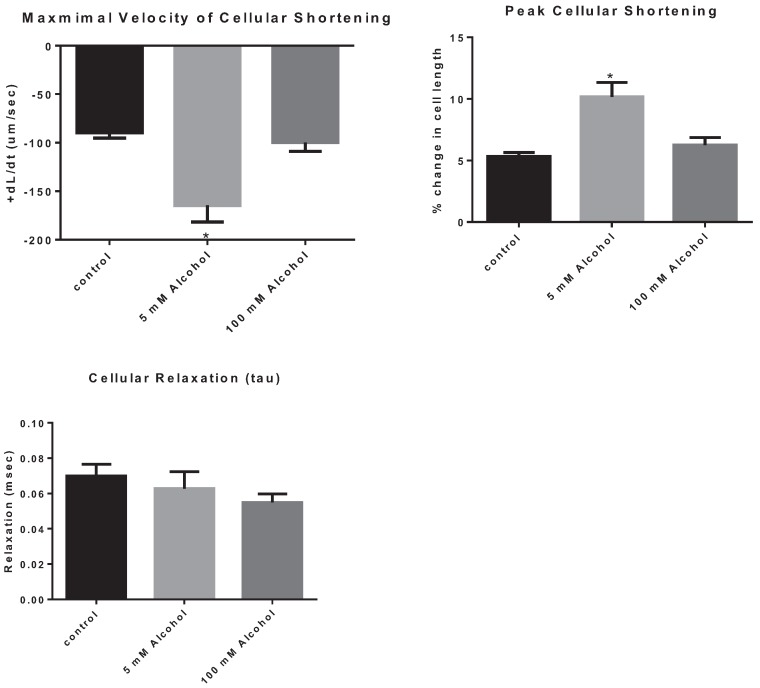
The effect of alcohol on cardiomyocyte contraction parameters, the maximal velocity of cellular contraction and cellular shortening, as well as on cellular relaxation. * *p* < 0.05 compared to control.

The effects of low and high alcohol on cellular contraction were tested on freshly isolated cardiomyocytes. Low dose of alcohol increased the speed of cellular shortening by 83.7% ± 0.24% (*p =* 0.0003) with LA subjects displaying a cellular shortening speed of, 164.6 ± 17.1 µm/s compared to 89.6 ± 5.6 µm/s for control subjects. In addition, LA increased peak cellular shortening by 92.4% ± 0.26% (*p =* 0.0001) with LA subjects displaying a peak of cellular shortening of 10.2% ± 1.2% compared to 5.3% ± 0.3% for control subjects (Figure 3). On the other hand, there was no significant effect of HA on the speed of contraction compared to control subjects, with a speed of contraction equal to 99.65 ± 9.3 µm/s (*p* > 0.05) for the HA subjects (Table 1). Also, the peak of cellular shortening was not significantly different between HA and control subjects, with HA subject displaying a peak cellular contraction of 6.2% ± 0.6% (*p* < 0.05). In addition, there was no significant effect of alcohol on cellular relaxation with either dose.

## 3. Discussion

It has been well established in scientific literature that chronic high alcohol exposure promotes apoptosis in cardiac cells [9], however low alcohol levels seem to confer certain protective effects on cardiomyocytes [12]. As such, we set out to ascertain the levels of positive caspase-3 events in the hearts of rats exposed to high alcohol, low alcohol, and no alcohol (control) by performing an immunohistochemical and cardiomyocyte contractility analysis.

Upon gross histological examination we observed a thinning of the left ventricular wall of high-alcohol rats compared to both control and low-alcohol rats. Immunohistochemical analysis suggests that elevated apoptosis may play a critical role in the left ventricular thinning associated with chronic high alcohol exposure. In contrast, the low alcohol exposure suggests a protective mechanism with a significant decrease in apoptotic events and increased contractility as evidenced by the increased speed and peak in cellular shortening.

High levels of alcohol can be particularly toxic to myocardial cells by severely damaging their structures. Chronic high alcohol can also cause intercalated disks to become fragile due to the breakdown of contractile elements [4]. In addition, it can cause the sarcoplasmic reticulum to become edematous and increase deposition of fats in the cell. [4,16,17]. These fat droplets can be surrounded by glycogen granules and are commonly found underneath the sarcolemma [18]. Also, the mitochondria in these cells tends to look swollen and markedly less dense with cristae [18]. Chronic alcohol has also been shown to decrease myocyte proliferation by up-regulating the expression of myostatin, a growth factor that is normally downregulated during tissue development [19]. Therefore, it is no surprise that increased levels of myostatin have been observed in the myocardium of individuals suffering from alcoholic cardiomyopathy [19]. Other studies have linked chronic alcohol consumption to a marked decrease in the myocardial expression of IGF-1 when compared to healthy controls. IGF-1 also plays a very important role in the proliferation and differentiation of cardiac myocytes. Furthermore, IGF-1 can serve as an anti-apoptotic factor since it can inhibit the activation of Bax and caspases, as well as DNA fragmentation [19]. Because of this we can see how an alcohol induced decrease in IGF-1 could negatively impact cardiomyocytes.

Most importantly though, based on studies conducted on rat cardiomyocytes we have learned that increasing quantities of alcohol can induce calcium release from the sarcoplasmic reticulum, causing its depletion and thus decreasing the myocardium’s ability to produce an appropriate contraction [20]. In addition to its detrimental effects on intracellular organelles and calcium homeostasis, chronic alcohol intake can also cause myocyte loss in the heart [3]. This very same loss of tissue can be the reason for the reduced myocardial function that manifests as ventricular dysfunction in patients with ACM [3]. The main process that is at the center of this phenomenon is apoptosis. Furthermore, cellular dysfunction would have an additional impact on the overall cardiac function. Accordingly we have found that LA enhanced cardiac function, which is associated with low caspase-3 levels. However, HA did not display a significant functional effect on the cellular level. This is in accordance to the recent work of Bebarova (2010) showing that the inhibitory effect of ethanol on sodium channels was only present at a toxic level of 500 mM or greater [21]. In addition, some apoptotic cells from HA hearts may not have survived the cellular isolation as non-apoptotic ones which could mask, to a certain extent, the detrimental effects of HA on cellular inotropy. Thus, we hypothesize that chronic high alcohol exposure will induce its detrimental cardiac effects through differential precipitation of apoptosis, whereas chronic exposure to low alcohol will enhance cardiac function through prevention of apoptosis.

An early molecular event that occurs in initiating apoptosis is the opening of the Bax/Bak channel, which results in the release of cytochrome c from the mitochondria [22]. Cytochrome c release results in the formation of the Apaf-1/caspase-9 apoptosome. This activates caspase-9 which in turn cleaves and activates caspase-3 and caspase-7. Caspase-3 then activates downstream caspases that play a major role in apoptosis. While caspase-9 activates both caspase-3 and caspase-7, prior studies have found that capsase-3 shows greater activation of downstream substrates compared to caspase-7. For example, it has been shown that caspase-3 cleaves pro-apoptotic molecules Bid and RIP1 in a much more efficient manner than caspase-7 [22]. In addition, caspase-3 is more efficient at propagating the caspase activation cascade [23]. This increased activity of caspase-3 makes it the major executioner caspase during the cell destruction phase of apoptosis [23]. Moreover, the multiple cellular apoptotic pathways converge at caspase-3, suggesting the reliable involvement of caspase-3 in apoptosis [23]. Due to these findings our study uses caspase-3 as a marker for apoptosis leading to the cardiomyocyte loss seen in ACM.

Conversely, caspase-3 can also participate in a pro-survival pathway by activating the anti-apoptotic kinase Akt. It does this by cleavage of the RasGAP protein [24]. Caspases also act on other substrates like p27^kip1^, Lyn, synphilin-1, and Rb in an anti-apoptotic capacity [24]. The effect of caspase-3 as an anti-apoptotic molecule occurs when caspase-3 is present in the cell at low levels [24]. At much higher caspase-3 levels such as those encountered in our experiment extensive cell death occurs. In our experiment, functionality data and caspase-3 expression levels indicate that there is heart failure in the rat cardiomyocytes, which is suggestive of the pro-apoptotic activity of caspase-3.

Experiments by Hajnóczky *et al.* [9], have shown the negative effects that mediators of apoptosis can have upon the hearts of transgenic mice. Conversely, upon providing caspase inhibitors to mice with failing hearts, the contractility of the heart chambers improved [9]. A study by Guan *et al.* [10] showed that cardiomyocytes exposed to high levels of alcohol exhibited irregular beating patterns, as well as cell death that was proportionate to ethanol concentration. This study also showed an increased presence of ROS in these cardiomyocytes. Moreover, caspase-3 levels increased and cytochrome c was found scattered in the cytoplasm rather than confined inside the mitochondria in cardiomyocytes exposed to high alcohol [10]. Another study noted enhanced levels of Bax and decreased levels of anti-apoptotic factors like Bcl-2 proteins in cardiomyocytes exposed to high alcohol environments [25]. It is critical that we point out that even a small increase in the rate of cell death in the human heart poses a significant threat because the majority of cardiomyocytes do not replicate seeing as they are terminally differentiated cells [9]. While the exact mechanisms by which chronic alcohol use triggers apoptosis is still being studied we do know that oxidative stress promotes the activation of the PTP, that is to say, it becomes more sensitive to the concentration of calcium [9]. This could very well be a contributor to the compromised permeability of the mitochondrial membrane during the initial steps of the pro-apoptotic pathway.

The data we obtained offers further evidence of the role of apoptosis as a potential mechanism of tissue loss in alcoholic cardiomyopathy. We were able to quantify a significant difference in the amounts of positive caspase-3 events seen in the hearts of rats exposed to low levels of alcohol *versus* high levels of alcohol. Based on our results we can postulate that the high number of positive caspase-3 events, a putative marker for apoptosis, are involved in the detrimental changes observed in the hearts of chronic alcoholics who exhibit cardiomyopathy. The apoptosis-induced loss of irreplaceable cardiac tissue would be responsible for the lower ejection fractions and higher end-diastolic volumes observed in heavy drinkers [4]. More importantly, this apoptotic loss of tissue would also be responsible for the wall thinning and ventricular dilation that ultimately leads to heart failure in these patients if abstinence is not commenced immediately. On the other hand, the latest clinical findings have shown that people with moderate exposure to alcohol have a better survival post-stroke than those that do not drink any alcohol at all. Therefore, we set out to determine the significance of apoptosis in mediating these peculiar cardioprotective effects of low alcohol exposure. Our experiments showed a smaller number of apoptotic events in the hearts of rats fed with low alcohol than those who had ingested no alcohol at all (control rats). Therefore, we allowed ourselves to conclude that chronic low alcohol exposure averts apoptosis and maintains the cardiac myofilament architecture and structural integrity of the ventricular wall. This would seem to enhance cellular capacity for survival, which is a key step in preventing the progression of heart failure. The mechanism by which these positive effects happen remains an enigma. We have recently eluded to the involvement of the PI3K/AKT pathway, with subsequent NRF2 phosphorylation [26].

## 4. Methods

Adult Wistar littermate male rats weighing between 150 g and 200 g from Harlan Laboratories (Madison, WI, USA) were used in this study (*n* = 6/group). Rats were allowed to acclimate to their new environment for one week after acquisition as they were kept under a 6:00 h to 18:00 h light cycle and were provided food and water *ad libitum*. Following acclimation, the subjects were placed on an isocaloric Lieber DeCarli liquid diet where the littermates were divided into groups: non-alcohol controls, low alcohol (5 mM) or high alcohol (100 mM) drinking groups for the next 4–5 months. All procedures were reviewed and approved by the Howard University Institutional Animal Care and Use Committee.

### 4.1. Histology

After a 4–5 months alcohol exposure period, animals were sacrificed and their hearts dissected and prepared for histological analysis [27,28]. Briefly, the subjects were deeply anaesthetized under isoflurane (0.5%) the heart was perfused with phosphate buffer solution (PBS; 0.1 M pH 7.4). The apex (lower 1/3 of the heart) was removed for Western blot analysis and the remaining heart tissue was post-fixed in 4% paraformaldehyde for 1 week at 4 °C. The hearts were then cryoprotected in graded sucrose solutions for an additional week (10%–30% sucrose), then frozen in isopentane at −65 °C and stored at −80 °C. Frozen heart samples were serially sectioned at a spacing of 1/5 at 50 µm in a Microm cryostat. One complete series was Nissl-stained with cresyl-violet for gross histological observations and the other series were banked in antigen preserve [27].

### 4.2. Immunohistochemistry

A single series of free-floating sections was removed from antigen preserve and washed three times with PBS [29] for batch immunohistochemistry [30]. Briefly, sections were blocked for thirty minutes in 3% normal horse serum (NHS) in PBS. Sections were then incubated overnight at room temperature in rabbit anti-caspase-3 (1:500; Cell Signaling, Danvers, MA, USA) in blocking solution. The following day, sections were washed in PBS and incubated for one hour and a half in a solution containing the horse anti-rabbit secondary antibody (1:200; Vector Laboratories, Burlingame, CA, USA). Following another set of PBS washes, the sections were incubated for 1.5 h in avidin-biotin (ABC Elite Kit; Vector Laboratories, Burlingame, CA, USA) and visualized with diaminobenzidine (DAB) as the chromogen (DAB Fast Tabs; Sigma-Aldrich, St. Louis, MO, USA). The sections were then mounted on gelatinized glass slides and allowed to dry overnight, dehydrated in graded alcohol solutions, cleared in xylenes and coverslipped with permount.

### 4.3. Image Analysis

Matched sections were imaged using an Olympus microscope at 20× magnification with a minimum of 3 sections per animal and 6 images/section/area (epicardium, endocardium, and myocardium). Positive caspase-3 events on each photograph were analyzed using ImageJ software (NIH, Bethesda, MD, USA, ImageJ 1.49, http://imagej.nih.gov/ij/). For the analysis each image was converted into a black and white 8-bit image and a number of features were changed in order to make the positive caspase-3 events easier to visualize and count. The contrast was enhanced to a saturated pixels level of 0.0%. Subsequently we subtracted the background using a rolling ball radius of 20 pixels. A threshold was established so that the program would exclude any caspase-3 events that were too light in color. The threshold used was 0–237. The size threshold was also adjusted so that only particles measuring above 250 pixels^2 were included in the count. Finally, the particles that remained were counted by the software and mapped on a schematic representation where they were highlighted in red. The analysis values were kept uniform for all images regardless of alcohol level or region in the heart.

### 4.4. Preparation of Rat Cardiomyocytes

Adult cardiomyocyte preparation was carried out as previously described (48). After anesthesia, the heart was rapidly removed, cannulated and retrogradely perfused (8 mL/min) via the aorta for 5 min (Langendorff technique) with a calcium containing standard Tyrode’s solution of the following composition (mM):NaCl (140), KCl (5.4), HEPES (10), MgCl_2_ (1), CaCl_2_ (1), d-glucose (10), 37 °C, pH balanced to 7.4 with NaOH or HCl. The heart was perfused with Ca^2+^ -free standard Tyrodes solution for 5 min. Thereafter the heart was digested with the same solution containing collagenase (type II, 0.55 mg/mL, Sigma-Aldrich, St. Louis, MO, USA) and protease (type XIV, 0.05 mg/mL, Sigma-Aldrich, St. Louis, MO, USA) for 8–9 min. Subsequently, the enzyme solution was removed by perfusing with Ca^2+^-free Tyrode’s solution. Myocytes were then filtered through a nylon mesh and re-suspended in Tyrode’s solution with incremental Ca^2+^ to a final concentration of 1 mM. Ca^2+^-tolerant cells were used immediately for acute studies.

### 4.5. Cellular Contraction and Relaxation

Freshly isolated cardiomyocytes were stimulated at 1 kHz using an external stimulator (Grass Instruments, Warwick, RI, USA). To determine cellular contraction and relaxation, the myocardial cells were imaged using a CCD video camera attached to the microscope and motion along a selected line segment was quantitated by a video motion detector system (IONOPTIX, Westwood, MA, USA). These measurements were recorded and analyzed using the Ionoptix IonWizard 6.3 software (IONOPTIX, Westwood, MA, USA). Acquisition of cellular contractions from a cardiomyocyte is performed over 1 min period which is averaged into a single average-twitch through an ensemble averaging process. After setting the baseline and the exponential asymptote on the average-twitch, cellular peak shortening is determined by the difference between cellular lengths before and during cardiomyocyte shortening. The velocities of contraction and relaxation are determined by separate exponential fittings of these events using quadratic iterations to calculate maximal and minimal change in length per unit time (+dL/dt and –dL/dt), respectively, using the IonWizard software.

### 4.6. Statistical Analysis

Analysis of group (treatment and area) was performed using an analysis of variance (ANOVA) followed by Student T-test using InStat statistical program.

## 5. Conclusions

Data presented here indicate that chronic low alcohol exposure averts apoptosis and maintains the cardiac myofilament architecture and structural integrity of the ventricular wall leading to improved contractility. Conversely chronic high alcohol consumption induced detrimental cardiac effects seems to be initiated by apoptosis leading to a thinning of the ventricular walls.
